# Drug interactions detected by a computer-assisted prescription system in primary care patients in Spain: MULTIPAP study

**DOI:** 10.1080/13814788.2021.1917543

**Published:** 2021-05-13

**Authors:** Eloísa Rogero-Blanco, Isabel Del-Cura-González, Mercedes Aza-Pascual-Salcedo, Francisca García de Blas González, Carmen Terrón-Rodas, Sergio Chimeno-Sánchez, Eva García-Domingo, Juan A. López-Rodríguez, group MULTIPAP

**Affiliations:** a Primary Health Care Center General Ricardos, Madrid, Spain; b URJC International Doctoral School, Interuniversity Program of Epidemiology and Public Health, Madrid, Spain; c Health Services Research on Chronic Patients Network (REDISSEC), Madrid, Spain; d Research Support Unit, Primary Care Management, Madrid, Spain; e Medical Specialties and Public Health Department, School of Health Sciences, University Rey Juan Carlos Alcorcón, Madrid, Spain; f Primary Care Direction, Sector Zaragoza III, Zaragoza, Spain; g Primary Care Management, Mendiguchia Carriche Health Center (Leganés, Madrid, Spain), Madrid, Spain; h Legazpi Health Center, Madrid, Spain; I Faculty of Health Sciences, URJC, Madrid, Spain; j Multiprofessional Teaching Unit of Family and Community Medicine of the Málaga/Guadalhorce Healthcare District, Malaga, Spain

**Keywords:** Drug interactions, polypharmacy, multimorbidity, primary care, drug therapy, computer-assisted

## Abstract

**Background:**

Drug interactions increase the risk of treatment failure, intoxication, hospital admissions, consultations and mortality. Computer-assisted prescription systems can help to detect interactions.

**Objectives:**

To describe the drug–drug interaction (DDI) and drug–disease interaction (DdI) prevalence identified by a computer-assisted prescription system in patients with multimorbidity and polypharmacy. Factors associated with clinically relevant interactions were analysed.

**Methods:**

Observational, descriptive, cross-sectional study in primary health care centres was undertaken in Spain. The sample included 593 patients aged 65–74 years with multimorbidity and polypharmacy participating in the MULTIPAP Study, recruited from November 2016 to January 2017. Drug interactions were identified by a computer-assisted prescription system. Descriptive, bivariate, and multivariate analyses with logistic regression models and robust estimators were performed.

**Results:**

Half (50.1% (95% CI 46.1–54.1)) of the patients had at least one relevant DDI and 23.9% (95% CI 18.9–25.6) presented with a DdI. Non-opioid–central nervous system depressant drug combinations and benzodiazepine–opioid drug combinations were the two most common clinically relevant interactions (10.8% and 5.9%, respectively). Factors associated with DDI were the use of more than 10 drugs (OR 11.86; 95% CI 6.92–20.33) and having anxiety/depressive disorder (OR 1.98; 95% CI 1.31–2.98). Protective factors against DDI were hypertension (OR 0.62; 95% CI 0.41–0.94), diabetes (OR 0.57; 95% CI 0.40–0.82), and ischaemic heart disease (OR 0.43; 95% CI 0.25–0.74).

**Conclusion:**

Drug interactions are prevalent in patients aged 65–74 years with multimorbidity and polypharmacy. The clinically relevant DDI frequency is low. The number of prescriptions taken is the most relevant factor associated with presenting a clinically relevant DDI.

KEY MESSAGES

- Drug interactions prevalence detected by a computer-assisted prescription system in multimorbidity and polypharmacy patients were high.
- Fifteen percent of the interactions were clinically relevant and affected 50% of the patients.
- Combinations of non-opioid drugs with potential depressant effects on the central nervous system was the most frequent interaction.

## Introduction

Polypharmacy brings with it an increase in drug interactions and adverse reactions. According to the APEAS report [1], 47.8% of adverse events in primary care are due to drugs, of which 3.5% are the consequence of drug interactions.

There are two main types of drug interaction: drug-drug interaction (DDI) and drug-disease interaction (DdI). Studies estimating the prevalence of DDI have shown a wide variability due to different classifications, patient profiles, and tools used. In Europe, with individuals over 20 years of age [2], the prevalence of DDI doubled from 5.8% to 13.1%. In Spanish primary care patients, DDI prevalence in people over 65 years of age ranged from 28% to 62.5% [3,4].

The existence of DDI increases the risk of treatment failure, intoxication, hospital admissions, number of consultations, and mortality [5]. Different systems have been developed to facilitate the detection of DDI. Alert programmes integrated into electronic prescription systems help the prescribing physician, though if they are repeated in excess, they may be ignored by professionals [6]. Computer-assisted prescription systems (CAPS) are another alternative that can improve the quality of prescribing by reducing medication errors. CheckTheMeds^®^ is a Spanish-language CAPS that offers a detailed analysis of pharmacological interactions and is used in the hospital setting as well as primary-care pharmacy services [7]. No studies analysed its use in the daily clinical practice of family doctors.

This study aimed to describe the drug-drug interaction (DDI) and drug-disease interaction (DdI) prevalence identified by a CAPS in patients with multimorbidity and polypharmacy. Factors associated with clinically relevant interactions were analysed.

## Methods

This was an observational, descriptive, cross-sectional, multicentre study in primary care, included in MULTIPAP STUDY [8] (trial registration number NCT 02866799). Patients between 65 and 74 years of age with multimorbidity (≥3 chronic diseases) and polypharmacy (≥5 drugs) who had visited their doctor at least once in the last year were studied. They were recruited between November 2016 and January 2017 by their physicians, who collected the variables in an interview and recorded them in an electronic data collection logbook. All patients signed written informed consent and the study was approved by the Committee of Ethics in Clinical Research of Aragon [8]. Given an expected percentage of potential severe DDI of 4% and the calculation of 95% confidence levels [9], it was estimated that the sample size of 593 would be sufficient to meet the study objective with a maximum precision error of 1.57%.

The interaction variables investigated were the numbers of DDI and DdI and their clinical relevance based on the UpToDate Lexicomp^®^ drug information database (https://www.uptodate.com/home/drugs-drug-interaction). Type D (‘consider therapy modification’) and X (‘avoid combination’) interactions were considered clinically relevant. Drug interactions were identified by a single researcher using CheckTheMeds^®^ (https://www.checkthemeds.com/). This CAPS uses various sources of information to analyse interactions. These include monthly bulletins and technical data sheets from the Spanish Agency of Medicines and Health Products, drug interaction studies, the U.S. Food and Drug Administration (FDA), Stockley’s drug Interactions, the American Geriatrics Society (AGS) 2019 Beers criteria Update Expert Panel, STOPP/START Version 2, Thorir D. Bjornsson et al.’s drug–drug interactions, the Thomson MICROMEDEX DRUGDEX^®^ System database, and Lexicomp^®^.

A descriptive analysis of the patient characteristics and all the DDI and DdI was performed according to clinical relevance. The qualitative variables are expressed as frequencies and percentages and the quantitative variables as mean (standard deviation) or median (interquartile range). The prevalence of DDI and DdI were estimated along with the corresponding 95% confidence intervals (CIs). The factors associated with clinically relevant DDI were analysed using a multiple logistic regression model with robust estimators to adjust confidence intervals to cluster sampling. The dependent variable was the presence of a clinically relevant DDI (X and D). The independent variables were those that in the bivariable analyses were statistically significant or were considered clinically important. Stata v14 was used for statistical analysis.

## Results

Sociodemographic and clinical characteristics of the patients have been published elsewhere [10]. Potential inappropriate prescribing has also been investigated [10]. A sub-analysis of drug interactions is presented below.

We analysed all interactions in 593 patients with 4386 prescribed drugs. A total of 3752 DDI were identified, of which 578 (15.4%) were clinically relevant, 517 (13.8%) being type D and 61 (1.6%) type X interactions. Of the 1768 DdI, 195 (11%) all clinically relevant ones were type D. From the patient standpoint, 97.1% of patients had some DDI, but only 297 (50.1%; 95% CI 46.1–54.1) had a clinically relevant DDI, including 279 (47%) type D and 54 (9.1%) type X interactions. The mean DDI number per patient was 1.1 (SD 1.5), with a maximum of eight. The most frequent were combinations of non-opioid drugs with potential depressant effects on the central nervous system (CNS) (10.8%), combinations of benzodiazepines with opioids (5.9%), and the joint use of amlodipine with simvastatin (5.2%). The most frequent type X DDI were the duplication of vitamin D analogues in 1.7% and the duplication of non-steroidal anti-inflammatory drugs (NSAIDs) (Table 1).

**Table 1.** Clinically relevant drug–drug interactions, types, effects, and frequencies.

Number of relevant DDIs (type D and/ or X) per patient		Number of patients (*n* = 297)	(%^a^)
1		137	23.1
2		59	9.9
3–4		78	13.2
≥ 5		23	3.9
Patients with at least 1 type D DDI Top 10 most frequent type D interactions	Effect	*n* = 279	47
Combination of drugs with CNS depressant effects (non-opioids)	Risk of CNS depressant effect	64	10.8
Benzodiazepines/opioids	Risk of deep sedation and respiratory depression	35	5.9
Amlodipine/Simvastatin	Risk of increased levels of simvastatin	31	5.2
Combination of high- and low-risk drugs for QT interval prolongation	Risk of QT segment prolongation	21	3.5
Duplication of benzodiazepines	Risk of sedation, falls and confusion	20	3.4
ACEI/ Allopurinol	Risk of skin reactions	16	2.7
Citalopram, escitalopram and cilostazol/CYP2C19 Inhibitors	Risk of QT segment prolongation	15	2.5
Triple whammy (ACEI or AIIRA/ diuretic/ NSAID)	Risk of renal failure	15	2.5
Insulins/ SGLT2 inhibitors	Risk of acidosis	14	2.3
Sulfonylureas/ DPP-IV Inhibitors	Risk of severe hypoglycaemia	13	2.2

^a^Percentage of the total number of patients studied (*n* = 593). DDI: drug–drug interaction; Type D: consider therapy modification; Type X: avoid combination; CNS: central nervous system; ACEI: angiotensin-converting enzyme inhibitor; AIIRA: angiotensin II receptor antagonist; PPI: proton pump inhibitor; NSAID: non-steroidal anti-inflammatory drug.

A total of 90.1% of patients presented some DdI, of which only 142 (23.9%; 95% CI 18.9–25.6) were relevant (type D). The mean number of relevant DdI per patient was 0.3 (SD 0.7), and there was a maximum of four simultaneous DdI per patient (Table 2). The use of long-acting β_2_ agonists in the presence of severe asthma, drug interactions affecting renal function, and the use of benzodiazepines in chronic obstructive pulmonary disease (5.5%, 4.9%, and 4.2%, respectively) were the most frequent (Table 2).

**Table 2.** Clinically relevant drug–disease interactions, types, effects, and frequencies.

Number of relevant DdIs per patient		Number of patients (*n* = 142)	(%^a^)
1		100	16.9
2		32	5.4
3-4		10	1.7
Patients with at least 1 type D DdI Top 10 most frequent type D interactions	Effect		
Use of long-acting β_2_ agonists in severe asthma	May worsen asthma	31	5.2
Interactions of various drugs that affect renal failure	Possibility of decreasing GF or renal toxicity	29	4.9
Use of benzodiazepines in COPD	Increased risk of respiratory depression	25	4.2
Use of beta-blockers in peripheral arterial disease	May worsen peripheral vascular disease	20	3.4
Use of COX-2 inhibitors in patients with high cardiovascular risk	Increases the risk of thrombotic events	8	1.3
Use of β_2_-agonists in patients with heart disease	May worsen heart function	6	1
Use of drugs that prolong the QT interval in patients with heart disease	Increased risk of prolonged QT interval in patients with heart disease	6	1
Use of benzodiazepines without antidepressants in anxiety/depression	Increased risk of increased self-injurious ideation	5	0.8
Use of vitamin D analogues in renal failure	May reduce the effectiveness of vitamin D analogues	5	0.8
Use of vitamin K analogues/various situations	Protein binding of the drug may be reduced	4	0.7

^a^Percentage of the total number of patients studied (*n* = 593). DdI: drug–disease interaction; Type D: consider therapy modification COPD: chronic obstructive pulmonary disease; GF: glomerular filtration.

The risk of presenting a clinically relevant DDI increased with the number of drugs (OR 11.86 (95% CI 6.92–20.33) for patients taking ≥10 drugs vs. 5–6 drugs). This risk decreased in patients with diabetes, high blood pressure (HBP), or ischaemic heart disease (Table 3).

**Table 3.** Factors associated with the presence of clinically relevant drug–drug interactions (type D and/or X) adjusted for age and sex.

	OR	95% CI	*p*
Number of drugs			
5–6 drugs	Reference		
7–9 drugs	2.83	1.89–4.25	<0.001
≥10 drugs	11.86	6.92–20.33	<0.001
Anxiety/depression	1.98	1.31–2.98	<0.001
Arterial hypertension	0.62	0.41–0.94	0.02
Diabetes	0.57	0.40–0.82	0.003
Ischaemic heart disease	0.43	0.25–0.74	0.002

Pseudo-*R*^2^: 0.1353; Akaike’s information criterion: 728.87; Bayesian information criterion: 768.34.

Type D interaction: consider therapy modification; Type X interaction: avoid combination.

## Discussion

### Main findings

In this cross-sectional study among 593 patients aged 65–74 years old with multimorbidity and polypharmacy recruited in Spanish primary health care centres, we identified drug interactions by a CAPS. Half of the patients had at least one relevant DDI and almost a quarter presented with a DdI. Non-opioid–central nervous system depressant drug combinations and benzodiazepine–opioid drug combinations were the two most common clinically relevant interactions. Using more than 10 drugs and having anxiety-depressive disorder were associated positively with DDI, whereas hypertension, diabetes and ischaemic heart disease were negatively associated with DDI.

### Interpretation

Comparing our results with the results from studies that have used different CAPS is difficult given the variability in the tools, definitions, and interaction classifications and because some studies have not provided data on severe interaction [3]. We present a synthesis of studies on the prevalence of interactions in the out-of-hospital setting in Supplementary Appendix 1.

The prevalence of DDI of any type that we found (97.1%) are higher than those reported in other studies (54.7–90.6%) [11, 12]. All patients in our study were patients with multimorbidity and polypharmacy, which may help explain these differences. However, these differences were lessened when we considered only clinically relevant DDI (type D or X). The study of outpatients in Saudi Arabia found type D DDI in 51.9% of patients [12], a value close to the 47% found in our study. Type X DDI were detected in 9.1% of our patients, much less than the 16.5% in the Saudi Arabian study [12].

As for the most frequent types of DDI, the DDI related to combinations of drugs with a risk of CNS depressant effects (non-opioids) affected 10.8% of our patients, a rate higher than the 5% found in a study conducted in Serbia [13]. These differences can be explained both by the high consumption of benzodiazepines in our patients (36.6%) and how we evaluated the drugs. In our case, a family doctor reviewed all the prescriptions, unlike the Serbian study, which was based on the exploration of databases. DDI related to the consumption of NSAIDs are frequent in many studies [3,14]. The combination of NSAIDs with diuretics and other drugs (‘triple whammy’) was one of the most frequent type D DDI (2.5%).

DdI has been much less studied. The prevalence of 23.9% obtained in our study is lower than the 64.1% described by Doubova et al. [9]. This difference may be due to the profile of drugs taken by patients (90.5% of their patients were taking NSAIDs vs 37.9%). DdI related to renal failure affected 4.9% of our patients, compared to 2.9% in the study by Doubova et al. [9]. This may be because those authors only described DdI between the use of NSAIDs and renal failure, while we also considered other drugs in such interactions.

The factor most strongly correlated with the number of relevant interactions is the number of drugs [14]. In our study, the DDI risk was increased up to 11-fold for those who took more than 10 drugs. A diagnosis of anxiety-depressive disorder also increased the risk of DDI, probably due to its association with increased consumption of CNS depressant drugs. Diseases such as DM, HBP, and ischaemic heart disease were associated with a lower risk of relevant DDI in our study. The treatments of these patients with prevalent, well-protocolised diseases are frequently reviewed, detecting interactions and avoiding them.

### Implications

The information obtained with CheckTheMeds^®^ is exhaustive, showing the professional all possible interactions, the adverse clinical consequences of the interactions, and the recommendation or action to follow. In general, most tools provide long lists of interactions without clinical relevance so that they would be useless in daily consultation. Defining the clinical relevance of interactions is essential, especially for patients with multimorbidity and polypharmacy.

CAPS can help detect interactions by facilitating their rapid and complete evaluation. They could support family doctors during consultation and would be more beneficial for decision-making if they prioritised interactions with clinical relevance. Future studies need to evaluate the potential utility of these CAPS by measuring relevant results in patients.

## Conclusion

Drug interactions are prevalent in patients aged 65–74 years with multimorbidity and polypharmacy. Combinations of non-opioid drugs with potential depressant effects on the central nervous system is one of the most frequent DDI. The clinically relevant DDI frequency is low. The number of prescriptions taken is the most relevant factor associated with presenting a DDI.

## Supplementary Material

Supplemental Material

## MULTIPAP group

**Lead authors for the MULTIPAP Study group:** Alexandra Prados Torres (Aragonese Institute of Health Sciences (IACS), IIS Aragón, Miguel Servet University Hospital, Spain) sprados. iacs@aragon.es, Juan Daniel Prados Torres (Multiprofessional Teaching Unit for Family and Community Care Primary Care District Málaga-Guadarhorce. Málaga) juand.prados.sspa@juntadeandalucia.es, Isabel del Cura (Research unit. Primary Health Care Management Madrid. Spain) isabel.cura@salud.madrid.org.

**Coordinating Committee:** José María Abad-Díez (Department of Health, Social Welfare and Family, Government of Aragon), Marta Alcaraz Borrajo (Subdirectorate General of Pharmacy and Health Products), Paula Ara Bardají (Aragonese Institute of Health Sciences (IACS), IIS Aragón, Miguel Servet University Hospital, Spain), Gloria Ariza Cardiel (Research unit. Primary Health Care Management Madrid. Spain), Mercedes Aza-Pascual-Salcedo (Primary Care Department, Aragonese Health Service.), Amaya Azcoaga Lorenzo (Pintores Primary Health Care Centre, Madrid, Spain), Ana Cristina Bandrés-Liso (Primary Care Department, Aragonese Health Service.), Mercedes Clerencia-Sierra (Unit of Social and Health Assessment, Miguel Servet University Hospital, Aragonese Health Service), Nuria García-Agua (Department of Pharmacology, Faculty of Medicine, Malaga University), Luis A. Gimeno Feliu (San Pablo Primary Health Care Centre, Aragon Health Service, Zaragoza, Spain), Antonio Gimeno-Miguel (Aragonese Institute of Health Sciences (IACS), IIS Aragón, Miguel Servet University Hospital, Spain), Ana I González González (Technical Support Unit, Primary Care Management, Madrid Health Service), Virginia Hernández Santiago (Ninewells Hospital & Medical School, Dundee, UK), Francisca Leiva Fernández (Multiprofessional Teaching Unit for Family and Community Care Primary Care District Málaga-Guadarhorce. Málaga), Ana M^a^ López-León (Alhaurín el Grande Health Centre, Malaga/Guadalhorce Sanitary District), Juan A López Rodríguez (Research unit. Primary Health Care Management Madrid. Spain), Cristina M Lozano Hernández (Research unit. Primary Health Care Management Madrid. Spain), María Isabel Márquez-Chamizo (Carranque Health Centre, Malaga/Guadalhorce Sanitary District.), Alessandra Marengoni (Department of Clinical and Experimental Sciences, University of Brescia, Brescia, Italy), Javier Marta-Moreno (Department of Neurology, University Hospital Miguel Servet, Aragonese Health Service.), Jesús Martín Fernández (Villamanta Primary Health Care Centre, Madrid, Spain), Angel Mataix SanJuan (Subdirección General de Farmacia y Productos Sanitarios), Carmina Mateos-Sancho (Ciudad Jardín Health Centre, Malaga/Guadalhorce Sanitary District), Christiane Muth (Institute of General Practice, Johann Wolfgang Goethe University, Frankfurt, Germany), Victoria Pico Soler (Torrero-LaPaz Health Centre, Zaragoza, Spain), Beatriz Poblador Plou (Aragonese Institute of Health Sciences (IACS), IIS Aragón, Miguel Servet University Hospital, Spain), Elena Polentinos Castro (Research unit. Primary Health Care Management Madrid. Spain), Antonio Poncel-Falcón (Primary Care Department, Aragonese Health Service.), Ricardo Rodríguez Barrientos (Research unit. Primary Health Care Management Madrid. Spain), José María Ruiz-San-Basilio (Coín Health Centre, Malaga/Guadalhorce Sanitary District), Mercedes Rumayor Zarzuelo (6 Centro de Salud Pública de Coslada, Área II Subdirección de Promoción de la Salud y Prevención), Luis Sánchez Perruca (Dirección Sistemas de Información, Gerencia Asistencial de Atención Primaria, Servicio Madrileño de Salud), Teresa Sanz Cuesta (Research unit. Primary Health Care Management Madrid. Spain), M^a^ Eugenia Tello Bernabé (El naranjo Primary Health Care Centre, Madrid, Spain.), José María Valderas Martínez (University of Exeter Medical School, Exeter, UK. 22Department), Rubén Vázquez-Alarcón (Vera Health Centre, AGS Norte de Almería).

### Clinical investigators in primary healthcare centres (PHC) MULTIPAP group

**Andalucía: PCHC Alhaurín el Grande** Javier Martín Izquierdo, Macarena Toro Sainz. **PCHC Carranque Andalucía):** M^a^ José Fernández Jiménez, Esperanza Mora García, José Manuel Navarro Jiménez. **PCHC Ciudad Jardín Andalucía)::** Deborah Gil Gómez, Leovigildo Ginel Mendoza, Luz Pilar de la Mota Ybancos, Jaime Sasporte Genafo.**PCHC Coín Andalucía):** M^a^ José Alcaide Rodríguez, Elena Barceló Garach, Beatriz Caffarena de Arteaga, M^a^ Dolores Gallego Parrilla, Catalina Sánchez Morales. **PCHC Delicia Andalucía):** M^a^ del Mar Loubet Chasco, Irene Martínez Ríos, Elena Mateo Delgado. **PCHC La Roca Andalucía):** Esther Martín Aurioles. **PCHC Limonar Andalucía):** Sylvia Hazañas Ruiz. **PCHC Palmilla Andalucía):** Nieves Muñoz Escalante. **PCHC Puerta Blanca Andalucía):** Enrique Leonés Salido, M^a^ Antonia Máximo Torres, M^a^ Luisa Moya Rodríguez, Encarnación Peláez Gálvez, José Manuel Ramírez Torres, Cristóbal Trillo Fernández. **PCHC Tiro Pichón Andalucı´a):** M^a^ Dolores García Martínez Cañavate, M^a^ del Mar Gil Mellado, M^a^ Victoria Muñoz Pradilla. **PCHC Vélez Sur Andalucía):** M^a^ José Clavijo Peña, José Leiva Fernández, Virginia Castillo Romero. **PCHC Victoria Andalucía):** Rafael Ángel Maqueda, Gloria Aycart Valdés, Miguel Domínguez Santaella, Ana M^a^ Fernández Vargas, Irene García, Antonia González Rodríguez, M^a^ Carmen Molina Mendaño, Juana Morales Naranjo, Catalina Moreno Torres, Francisco Serrano Guerra.

**Aragón: PCHC Alcorisa** (Alcorisa): Carmen Sánchez Celaya del Pozo. **PCHC Delicias Norte** (Zaragoza): José Ignacio Torrente Garrido, Concepción García Aranda, Marina Pinilla Lafuente, M^a^ Teresa Delgado Marroquín. **PCHC Picarral** (Zaragoza): M^a^ José Gracia Molina, Javier Cuartero Bernal, M^a^ Victoria Asín Martín, Susana García Domínguez. **PCHC Fuentes de Ebro** (Zaragoza): Carlos Bolea Gorbea. **PCHC Valdefierro** (Zaragoza): Antonio Luis Oto Negre. **PCHC Actur Norte** (Zaragoza): Eugenio Galve Royo, M^a^ Begoña Abadía Taira. **PCHC Alcañiz** (Alcañiz): José Fernando Tomás Gutiérrez. **PCHC Sagasta— Ruiseñores** (Zaragoza): José Porta Quintana, Valentina Martín Miguel, Esther Mateo de las Heras, Carmen Esteban Algora. **PCHC Ejea** (Ejea de los Caballeros): M^a^ Teresa Martín Nasarre de Letosa, Elena Gascón del Prim, Noelia Sorinas Delgado, M^a^ Rosario Sanjuan Cortés. **PCHC Canal Imperial—Venecia** (Zaragoza): Teodoro Corrales Sánchez. **PCHC Canal Imperial—San José Sur** (Zaragoza): Eustaquio Dendarieta Lucas. **PCHC Jaca** (Jaca): M^a^ del Pilar Mínguez Sorio. Virginia López Cortés. **PCHC Santo Grial** (Huesca): Adolfo Cajal Marzal.

**Madrid. PCHC Mendiguchía Carriche** (Leganés): Eduardo Díaz García, Juan Carlos García Álvarez, Francisca García De Blas González, Cristina Guisado Pérez, Alberto López García Franco, M^a^ Elisa Viñuela Benitez. **PCHC El Greco** (Getafe): Ana Ballarín González, M^a^ Isabel Ferrer Zapata, Esther Gómez Suarez, Fernanda Morales Ortiz, Lourdes Carolina Peláez Laguno, José Luis Quintana Gómez, Enrique Revilla Pascual. **PCHC Cuzco** (Fuenlabrada): M Ángeles Miguel Abanto. **PCHC El Soto** (Móstoles): Blanca Gutierrez Teira. **PCHC Genera Ricardos** (Madrid): Francisco Ramón Abellán López, Carlos Casado Álvaro, Paulino Cubero González, Santiago Manuel Machín Hamalainen, Raquel Mateo Fernández, M^a^ Eloisa Rogero Blanco, Cesar Sánchez Arce. **PCHC Ibiza** (Madrid): Jorge Olmedo Galindo. **PCHC Las Américas** (Parla): Claudia López Marcos, Soledad Lorenzo Borda, Juan Carlos Moreno Fernández, Belén Muñoz Gómez, Enrique Rodríguez De Mingo. **PCHC M^a^ Ángeles López** (Leganés): Juan Pedro Calvo Pascual, Margarita Gómez Barroso, Beatriz López Serrano, M^a^ Paloma Morso Peláez, Julio Sánchez Salvador, Jeannet Dolores Sánchez Yépez, Ana Sosa Alonso. **PCHC M^a^ Jesús Hereza** (Leganés): M^a^ del Mar A^´^lvarez Villalba. **PCHC Pavones** (Madrid): Purificación Magán Tapia. **PCHC Pedro Laín Entralgo** (Alcorcón): M^a^ Angelica Fajardo Alcántara, M^a^ Canto De Hoyos Alonso, M^a^ Aránzazu Murciano Antón. **PCHC Pintores** (Parla): Manuel Antonio Alonso Pérez, Ricardo De Felipe Medina, Amaya Nuria López Laguna, Eva Martínez Cid De Rivera, Iliana Serrano Flores, M^a^ Jesús Sousa Rodríguez. **PCHC Ramón y Cajal** (Alcorcón): M^a^ Soledad Núñez Isabel, Jesús M^a^ Redondo Sánchez, Pedro Sánchez Llanos, Lourdes Visedo Campillo.
